# IgE Immune Complexes Stimulate an Increase in Lung Mast Cell Progenitors in a Mouse Model of Allergic Airway Inflammation

**DOI:** 10.1371/journal.pone.0020261

**Published:** 2011-05-19

**Authors:** Joakim S. Dahlin, Martin A. Ivarsson, Birgitta Heyman, Jenny Hallgren

**Affiliations:** Department of Medical Biochemistry and Microbiology, Uppsala University, Uppsala, Sweden; University Hospital Freiburg, Germany

## Abstract

Mast cell numbers and allergen specific IgE are increased in the lungs of patients with allergic asthma and this can be reproduced in mouse models. The increased number of mast cells is likely due to recruitment of mast cell progenitors that mature *in situ*. We hypothesized that formation of IgE immune complexes in the lungs of sensitized mice increase the migration of mast cell progenitors to this organ. To study this, a model of allergic airway inflammation where mice were immunized with ovalbumin (OVA) in alum twice followed by three daily intranasal challenges of either OVA coupled to trinitrophenyl (TNP) alone or as immune complexes with IgE-anti-TNP, was used. Mast cell progenitors were quantified by a limiting dilution assay. IgE immune complex challenge of sensitized mice elicited three times more mast cell progenitors per lung than challenge with the same dose of antigen alone. This dose of antigen challenge alone did not increase the levels of mast cell progenitors compared to unchallenged mice. IgE immune complex challenge of sensitized mice also enhanced the frequency of mast cell progenitors per 10^6^ mononuclear cells by 2.1-fold. The enhancement of lung mast cell progenitors by IgE immune complex challenge was lost in FcRγ deficient mice but not in CD23 deficient mice. Our data show that IgE immune complex challenge enhances the number of mast cell progenitors in the lung through activation of an Fc receptor associated with the FcRγ chain. This most likely takes place via activation of FcεRI, although activation via FcγRIV or a combination of the two receptors cannot be excluded. IgE immune complex-mediated enhancement of lung MCp numbers is a new reason to target IgE in therapies against allergic asthma.

## Introduction

The lungs from patients with allergic asthma and mice with antigen-induced allergic lung inflammation share several common features such as increased numbers of Th2-cells, eosinophils and mast cells as well as increased levels of antigen-specific IgE [1], [2], [3]. Mast cells originate from bone marrow derived mast cell progenitors (MCp) that upon entry to the peripheral organs mature into mast cells [4]. Naïve mice and mice sensitized with intraperitoneal injection of antigen have few MCp in their lungs, but antigen aerosol challenge induces MCp recruitment and increases both frequency and the total number of MCp per lung [5]. For recruitment to occur, the MCp need to migrate from the blood vessels through the lung endothelium. This process is dependent on expression of α4-integrins on the MCp interacting with endothelial VCAM-1 [5]. In addition, although MCp recruitment is regulated by CXCR2 and CCL2/CCR2, the chemokine receptors need to be expressed by the lung stroma cells rather than on the MCp for optimal recruitment to lung [3], [6]. Furthermore, MCp recruitment is regulated by IL-9, most likely produced by NKT-cells [7].

The importance of allergen-specific IgE production in allergic asthma has been widely appreciated through the recent success with the anti-IgE monoclonal antibody, omalizumab [8]. This antibody targets the Fc-binding part of IgE thereby hindering the binding of IgE and IgE immune complexes to IgE-receptors and leads to an attenuation of asthma exacerbations and symptoms. Further, genetic analyses reveal an association between IgE levels and asthma severity [9], [10]. Studies in mice have shown that IgE and IgE immune complexes can bind to several activating receptors. Most well known is the high affinity receptor for IgE, FcεRI, expressed mainly on mast cells and basophils. Besides activating mast cells and basophils leading to degranulation and release of proinflammatory mediators, IgE also functions as a survival factor for these cells [11]. FcγRIII is another receptor that bind IgE immune complexes of certain allotypes with low affinity [12]. A more recently described receptor that binds IgE immune complexes is FcγRIV, found on macrophages, dendritic cells and polymorphonuclear cells [13], [14]. Like FcγRIII and FcεRI, FcγRIV is dependent on the common FcRγ-chain for signaling and is thought to synergize with FcεRI in the lung to mediate the IgE immune complex induced infiltration of Mac1^+^ Gr1^+^ polymorphonuclear cells seen in bronchoalveolar lavage [14]. In addition, IgE and IgE immune complexes bind to CD23, a C-type lectin expressed by B-cells and follicular dendritic cells. This receptor is required for the IgE-mediated enhancement of antibody and CD4^+^ T-cell responses seen after primary intravenous immunizations [15], but is also thought to negatively regulate the immune response [16]. Thus, there are several receptors that IgE immune complexes could utilize to stimulate the immune response.

Because enhanced antigen specific IgE and enhanced numbers of lung mast cells is a shared phenomenon in human asthma and in mouse models for this disease, we hypothesized that formation of IgE immune complexes locally in the lung enhances migration of MCp into the lung. To test this, OVA sensitized mice were given an intranasal challenge with either OVA-TNP alone or together with IgE-anti-TNP as an immune complex. Using a model of allergic airway inflammation, we demonstrate for the first time that IgE immune complex challenge stimulated MCp recruitment to the lung when compared to challenge with same dose antigen alone. The IgE immune complex stimulated MCp recruitment was dependent on the engagement of an Fc receptor that use the common FcRγ chain for signaling, most likely FcεRI.

## Materials and Methods

### Ethics statement

All animal experiments were approved by Uppsala animal research ethics committee (protocol number C116/7 and C3/10). The mice were bred and maintained in the animal facilities at the National Veterinary Institute (Uppsala, Sweden). Skilled personnel under the supervision of the veterinarian in charge routinely observed the health status of the mice.

### Animals

6–14 week old male BALB/c, CD23^−/−^ [17] backcrossed to BALB/c for 10 generations, and FcRγ^−/−^ [18] backcrossed to BALB/c for 12 generations were used.

### Reagents

OVA grade V purchased from Sigma-Aldrich (St Louis, MO, USA) was used for sensitization and for coupling to TNP (picrylsulfonic acid/hydrate) (Sigma-Aldrich). TNP was coupled to OVA in 0.28 M cacodylate buffer, pH 6.9 at room temperature. After 80 min incubation, the reaction was stopped by adding excess of glycyl-glycine (1 mg/ml; Merck, Darmstadt, Germany). The formed OVA-TNP was dialyzed against PBS, sterile filtered and kept at +4°C. The number of TNP residues per OVA was obtained by measuring the absorbance at 280 nm and 340 nm, in accordance with [19]. A ratio of 2.3 TNP∶OVA was used. Monoclonal IgE-anti-TNP was obtained from the IGELb4 hybridoma producing murine IgE-anti-TNP [20]. IGELb4 were cultured in DMEM supplemented with 5% heat-inactivated FCS, 10 mM HEPES, 100 U/ml penicillin, 100 µg/ml streptomycin, 2 mM L-glutamine, 1 mM sodium pyruvate, 50 µM 2-mercapto ethanol (all from Sigma-Aldrich). IgE was purified by affinity chromatography on a Sepharose column conjugated with monoclonal rat anti-mouse *kappa* (187.1.10) and eluted with 0.1 M glycine-HCl buffer, pH 2.8 [21]. The solution was dialyzed to PBS, sterile filtered, quantified by reading the absorbance at 280 nm assuming that an absorbance of 1.5 equals 1 mg/ml IgE and stored at −20°C.

### OVA sensitization and challenge protocol

**Figure 1:**
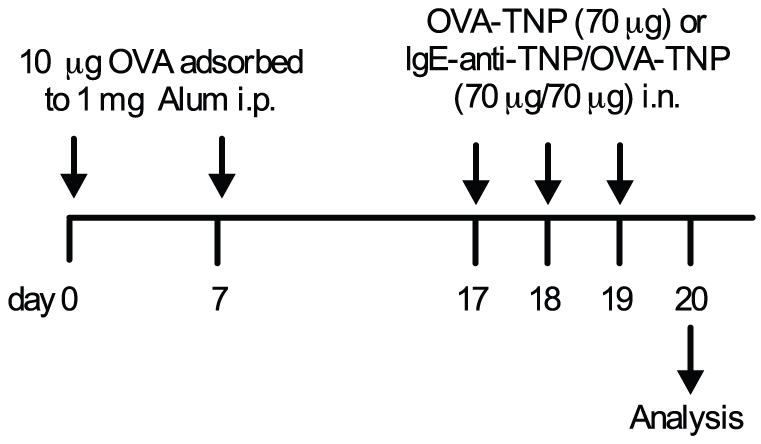
An outline of the experimental protocol. All mice were sensitized day 0 and day 7 with i.p. injections of 10 µg OVA adsorbed to 1 mg alum. Day 17–19 the mice were challenged intranasally (i.n.) with either 70 µg OVA-TNP alone or together with 70 µg IgE-anti-TNP. Mice were analyzed 24 h after challenge (see materials and methods).

Mice were sensitized on day 0 and day 7 with 10 µg OVA grade V (Sigma-Aldrich) adsorbed to 1 mg alum (Pierce, Thermo Scientific, Rockford, USA) in 200 µl sterile PBS. Day 17–19 mice were anesthesized with 50 µl solution consisting of Hypnorm (Vetapharma Ltd, Leeds, UK) and Dormicum (Roche, Basel, Switzerland) given subcutaneously. This solution was prepared by diluting Hypnorm 1∶1 with sterile water and Dormicum 1∶1 with sterile water separately and then mixing them together (1∶1). Anesthesized mice were given daily intranasal doses with either 70 µg OVA-TNP alone in sterile PBS or together with 70 µg IgE-anti-TNP (Figure 1). Both solutions were prepared in a total volume of 30 µl. In some experiments OVA sensitized mice were instead challenged with 1% OVA in PBS as aerosol using a PARI nebulizer (Starnberg, Germany) for 30 min daily during day 17–19.

### Preparation of mononuclear cells and mast cell progenitor quantification

On day 20, mice were killed by an overdose of isoflurane (Schering Plough A/S, Farum, Denmark) and the lungs were perfused with 10 ml of sterile PBS administered via the right ventricle. Lungs and spleens were harvested into ∼10 ml of complete RPMI (RPMI 1640 containing 100 U/ml penicillin, 100 µg/ml streptomycin, 10 µg/ml gentamicin, 2 mM L-glutamine, 10 mM HEPES, 0.1 mM non-essential amino acids, 1 mM sodium pyruvate, 50 µM 2-mercaptoethanol and 10% heat-inactivated FCS (all from Sigma-Aldrich)). In one experiment, the mice were anesthetized and bronchoalveolar lavage was performed by flushing the lungs twice with 0.5 ml PBS.

The obtained lungs were finely chopped with scalpels and transferred to 50 ml plastic tubes with 10 ml of complete RPMI 1640 plus 1 ml of ∼1830 units of collagenase type IV (Gibco, Paisley, Scotland, UK or Worthington, Lakewood, NJ, USA). The lung homogenates were incubated for ∼20 min at 37°C. The released cells were harvested whereas the undigested tissue pieces were subjected to another enzymatic digestion (for a total of three digestions). The cells from the three digestions were pelleted, resuspended in 44% Percoll (Sigma-Aldrich) and underlayed with a 67% Percoll layer. After spinning at 400× *g* for 20 min at 4°C, lung mononuclear cells (MNC) were harvested from the 44/67% Percoll interface. The MNC obtained from two Percoll gradients per mouse lung were pooled and washed in complete RPMI 1640. The total number of viable MNC was determined by trypan blue dye exclusion on a hemacytometer. The cells were serially 2-fold diluted to eight concentrations beginning at 20,000 cells/well in complete RPMI and 100 µl of each dilution was added to eight wells of two sterile 96-well flat-bottomed tissue culture plates per mouse lung. Thereafter, each well received 100 µl of gamma-irradiated (30 Gy) splenic feeder cells plus IL-3 (40 ng/ml) and SCF (40 ng/ml). Murine IL-3 was obtained as supernatants from the X-63 B-cell line [22] and quantified by ELISA. Recombinant SCF was purchased from Peprotech (#250-03, Rocky Hill, NJ, USA). After 10–12 days of incubation in humidified 37°C incubators with 5% CO_2_, wells containing mast cell colonies were counted with an inverted microscope. After culture, only mast cell colonies appeared in the plates. They were distinguished from the feeder matrix as small-medium sized round cells in large colonies [5], [23], [24]. The total number of lung MCp/mouse was derived by multiplying the concentration of MCp (MCp/10^6^ MNC) by the total number of MNC obtained from each mouse lung.

### Flow cytometry

Lung MNC or cells from bronchoalveolar lavage were washed in FACS buffer (2% FCS in PBS pH 7.4) and fluorescence staining was performed at 4°C in 100 µl FACS buffer for 30 min. After pre-incubation with Fc-block (2.4G2, BD Bioscience, Franklin Lakes, NJ, USA), the following antibodies were used to detect CD3^+^ and CD3^+^/CD4^+^ T cells and CD19^+^ B cells: Alexa 488 conjugated hamster anti-mouse CD3 (500-A2, Caltag Laboratories, Caltag-Medsystems Ltd, Buckingham, UK), PE conjugated rat anti-mouse CD4 (L3T4, BD Bioscience) and FITC conjugated rat anti-mouse CD19 (1D3, BD Bioscience). Dendritic cells were identified as CD11c^+^, MHC-II^hi^ cells using APC conjugated hamster anti-mouse CD11c (HL3, BD Bioscience) and PE conjugated mouse anti-mouse I-A^d^ (AMS-32.1, BD Bioscience). PE-Cy7 conjugated hamster anti-mouse CD11c (N418, eBioscience, Hatfield, UK), FITC conjugated rat anti-mouse CD45 (30-F11, BD Bioscience) and PE conjugated rat anti-mouse SiglecF (E50-2440, BD Bioscience) along with matched isotype controls were used to detect eosinophils in bronchoalveolar lavage. Cells were analyzed using a FACScan or a LSRII (BD Bioscience) cytometer and analyzed using the FlowJo software (Tree Star Inc, Ashland, OR, USA).

### Statistics

As the method used for quantifying lung MCp is very time consuming we were unable to perform individual experiments with enough animals in each group to obtain statistics. Thus, the values obtained from analyses of each individual mouse in all experiments were used and the groups were compared using a two-way ANOVA. Before the analysis the values were transformed to a Gaussian distribution by calculating the natural logarithm of each value. The p value for the interaction term was greater than 0.05 in all analyses. Differences were considered statistically significant if p<0.05. Eosinophils were quantified in one experiment and these groups were compared using an unpaired Student's t-test. The statistics were calculated using GraphPad Prism 5.0b.

## Results

### IgE immune complex challenge enhance the lung mast cell progenitor number compared to the same dose antigen alone

**Figure 2:**
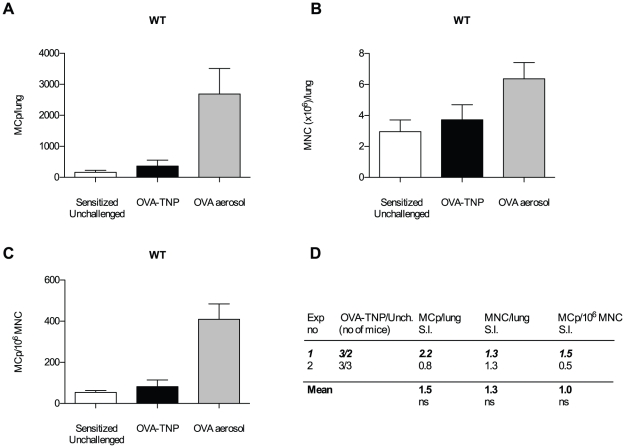
Intranasal challenge with OVA-TNP does not enhance the lung MCp number over basal levels. (A–C) One experiment showing the quantification of MCp and MNC from wild type (WT) mice sensitized with OVA/alum and challenged with either OVA-TNP alone, OVA aerosol or left unchallenged. The error bars shown are SEM. (D) A comparison of the OVA-TNP challenged and the unchallenged group from the two experiments performed expressed as a stimulation index (S.I.) where the numbers represent the mean number of MCp/lung, MNC/lung or MCp/10^6^ MNC per experiment of sensitized mice challenged with OVA-TNP divided by the mean number obtained from sensitized mice left unchallenged. The mean of the S.I. from the experiments is given in bold text at the bottom. The experiment shown in (A–C) is indicated in bold italics. There was no statistical difference (ns) between the OVA-TNP challenged and the unchallenged mice in the tested parameters using a two-way ANOVA from a comparison of all individual mice from each group from the two experiments.

Previously, the same sensitization protocol as in the present investigation was used in studies of antigen-induced MCp recruitment to lung, but the mice were challenged with 1% OVA-aerosol (8 ml of 10 mg/ml) for 30 min a day [5] instead of 70 µg OVA-TNP intranasally. To establish how efficient 70 µg OVA-TNP administered intranasally is to induce higher numbers of lung MCp as compared to only sensitized (not challenged) or OVA aerosol challenged mice, we analyzed the lung MCp in these groups (Figure 2). As expected from earlier studies, sensitized mice challenged with 1% OVA aerosol had high numbers of lung MCp, 2687±826 MCp/lung (Figure 2A) and 409±76 MCp/10^6^ MNC (Figure 2C). OVA sensitization and aerosol challenge thus induced a 10-fold increase in total lung MCp and a 5-fold increase in MCp frequency in the lungs as compared to sensitized mice challenged intranasally with OVA-TNP and unchallenged mice (p<0.05). In contrast, sensitized mice challenged with OVA-TNP alone had the same frequency and total amount of lung MCp as sensitized mice left unchallenged (Figure 2D). As described previously, OVA sensitized and naïve mice have similar low basal homing of MCp to lung [5], [25]. Thus, 70 µg OVA-TNP alone is unable to induce an enhancement of lung MCp over the basal homing.

**Figure 3:**
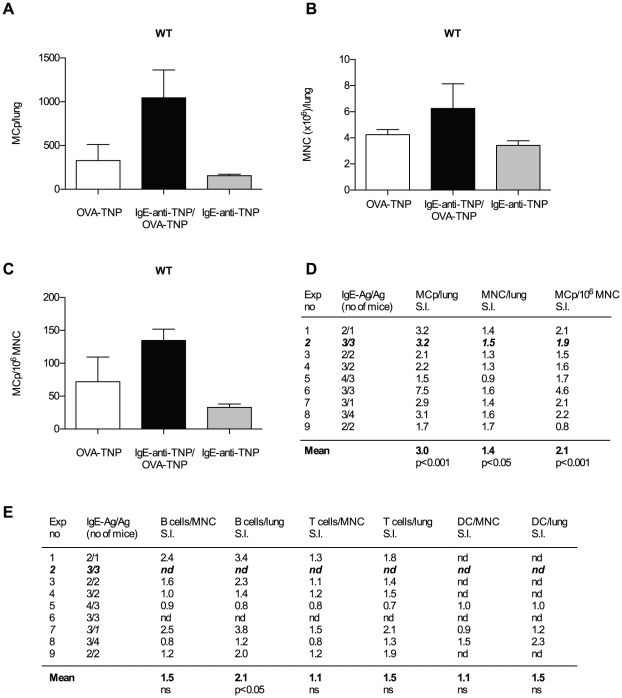
Challenge with IgE immune complex enhance lung MCp numbers compared to antigen alone. (A–C) A representative experiment showing the quantification of MCp and MNC from wild type (WT) mice sensitized with OVA/alum and challenged with OVA-TNP, IgE-anti-TNP/OVA-TNP or IgE-anti-TNP alone. The error bars shown are SEM. (D) A summary of all nine experiments performed expressed as a stimulation index (S.I.) where the numbers represent the mean number of MCp/lung, MNC/lung or MCp/10^6^ MNC per experiment of sensitized mice challenged with IgE-anti-TNP/OVA-TNP divided by the mean number obtained from sensitized mice challenged with OVA-TNP alone for each experiment. (E) A summary of all the experiments where B-cells, T-cells and dendritic cells (DC) were analyzed with flow cytometry. In some experiments flow cytometry was not done (nd). The mean of the S.I. from all experiments is given in bold text at the bottom. The experiment shown in (A–C) is indicated in bold italics. The p-values shown are derived from two-way ANOVA from a comparison of all individual mice in each group from the nine experiments.

To study the effects of IgE immune complexes on the number of MCp in the lung, OVA sensitized BALB/c mice were challenged with either OVA-TNP alone or IgE-anti-TNP/OVA-TNP immune complexes intranasally during three consecutive days. The numbers of MCp as well as MNC in the two groups were compared. One representative out of the nine independent experiments performed is shown in Figure 3A–C. Here, sensitized mice given OVA-TNP challenge alone had 327±185 MCp per lung whereas IgE-anti-TNP/OVA-TNP challenge enhanced the number of MCp 3.2-fold to 1046±317 MCp per lung (Figure 3A). IgE alone did not enhance the MCp numbers in the lung (Figure 3A). Looking at all nine experiments, 3.0 times more lung MCp were elicited in sensitized IgE-anti-TNP/OVA-TNP challenged mice compared to OVA-TNP challenged animals (p<0.001, Figure 3D left column). We noted a small enhancement (1.4-fold) in the number of MNC in IgE-anti-TNP/OVA-TNP challenged compared to OVA-TNP challenged mice (Figure 3B, D), suggesting that other cell populations were also influenced. However, the specific increase in MCp/10^6^ MNC was 2.1 times higher in sensitized mice challenged with IgE-anti-TNP/OVA-TNP than in mice challenged with OVA-TNP (Figure 3C, D). Thus, IgE-anti-TNP/OVA-TNP immune complexes do not just increase the number of MNC, but stimulate an increase in lung MCp specifically.

### Effects of IgE immune complex challenge on other cell populations in the lungs

Since the MNC numbers were increased, albeit only 1.4-fold, in sensitized mice challenged with IgE-anti-TNP/OVA-TNP, flow cytometry analyses of the residual MNC left after setting up the assay to quantify MCp was performed. We analyzed the percentage and total number of B-cells (CD19^+^), T-cells (both CD3^+^ and CD3^+^/CD4^+^) as well as dendritic cells (CD11c^+^, MHCII^hi^). As shown in Figure 3E, there was an increase in the total number, but not in the frequency, of B-lymphocytes and a tendency towards an increased total number of CD3^+^ T-cells. There was also a tendency towards increased total numbers of CD4^+^ T-helper cells (results not shown). Dendritic cells were analyzed in three experiments but we could not detect any differences between the treatment groups (Figure 3E).

Since a paper by Zuberi et al [26] demonstrated using a similar protocol that eosinophil numbers were increased in bronchoalveolar lavage fluid in sensitized mice challenged with IgE immune complexes, one experiment where bronchoalveolar lavage was analyzed for eosinophils (CD11c^−^, CD45^+^, Siglec- F^+^) was performed. Sensitized and IgE-anti-TNP/OVA-TNP challenged mice had 37±6% eosinophils and 37957±10343 in total whereas sensitized mice given the same dose OVA-TNP alone had less eosinophils (17±7%, p = 0.07; 7078±3891 in total number, p<0.05).

### CD23 deficient mice have normal number of lung mast cell progenitors after IgE immune complex challenge

**Figure 4:**
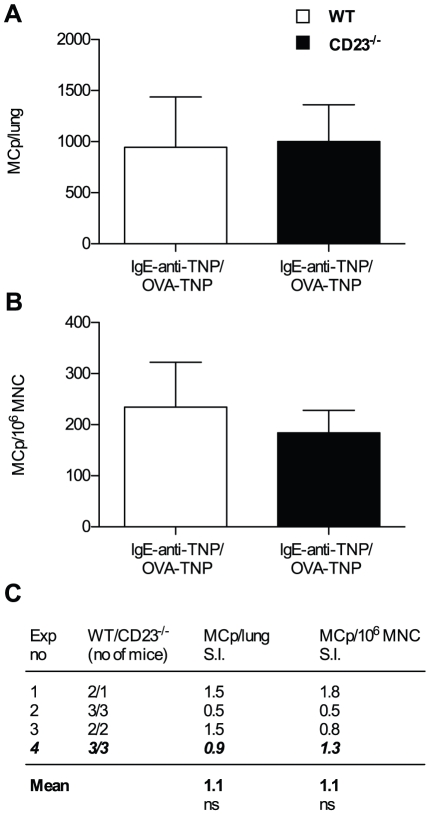
IgE immune complex challenge induces similar lung MCp levels in CD23^−/−^ as in wild type. (A–B) A representative experiment showing the quantification of MCp from wild type (WT) or CD23^−/−^ mice sensitized with OVA/alum and challenged with IgE-anti-TNP/OVA-TNP. The error bars shown are SEM. (C) A summary of all the four experiments performed expressed as a stimulation index (S.I.) where the numbers represent the mean number of MCp/lung or MCp/10^6^MNC per experiment of sensitized WT mice challenged with IgE-anti-TNP/OVA-TNP divided by the mean number obtained from CD23^−/−^ mice treated in parallel. The mean of the S.I. from all experiments is given in bold text at the bottom. The representative experiment shown in (A–B) is indicated in bold italics. There was no statistical difference (ns) in the tested parameters using a two-way ANOVA from a comparison of all individual mice from each group from the four experiments.

CD23 is one candidate receptor for mediating biological effects of IgE immune complexes. However, OVA sensitized and IgE-anti-TNP/OVA-TNP challenged CD23^−/−^ and wild type mice had similar numbers of MCp in their lungs (CD23^−/−^: 1002±359 MCp per lung versus WT: 944±493) (Figure 4A). Further, there were no differences in the frequency of MCp/10^6^ MNC (Figure 4B) and both strains had similar MNC yields (results not shown). Altogether, four experiments comparing IgE-anti-TNP/OVA-TNP challenge of OVA sensitized CD23^−/−^ and BALB/c mice resulted in stimulation indices close to 1 (Figure 4C). Thus, the stimulating effect of IgE immune complexes on lung MCp numbers is not mediated by CD23.

### IgE immune complex-induced enhancement of lung mast cell progenitors is lost in FcRγ deficient mice

**Figure 5:**
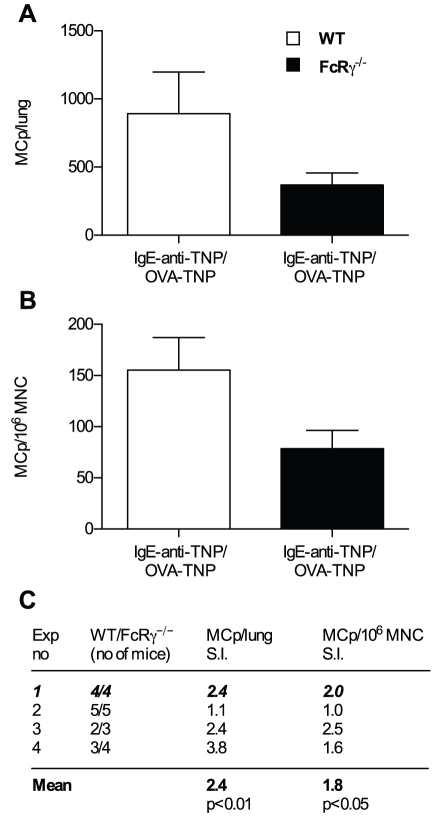
FcRγ-chain deficient mice have a reduced number of lung MCp after IgE immune complex challenge. (A–B) A representative experiment showing the quantification of MCp from wild type (WT) or FcRγ^−/−^ sensitized with OVA/alum and challenged with IgE-anti-TNP/OVA-TNP (C) A summary of all the four experiments performed expressed as a stimulation index (S.I.) where the numbers represent the mean number of MCp/lung or MCp/10^6^ MNC of sensitized WT mice challenged with IgE-anti-TNP/OVA-TNP divided by the mean number obtained in FcRγ^−/−^ mice treated in parallel. The mean of the S.I. from all experiments is given in bold text at the bottom. The representative experiment shown in (A–B) is indicated in bold italics. The p-values shown are derived using a two-way ANOVA from a comparison of all individual mice from each group from the four experiments.

**Figure 6:**
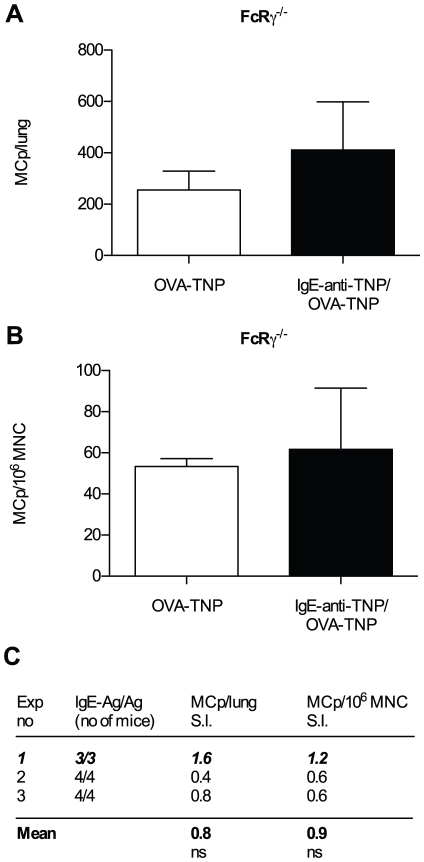
IgE immune complex-induced enhancement of lung MCp is dependent on an FcRγ chain associated receptor. (A–B) A representative experiment showing the quantification of MCp from or FcRγ^−/−^ sensitized with OVA/alum and challenged with either IgE-anti-TNP/OVA-TNP or OVA-TNP alone (C) A summary of all the three experiments performed expressed as a stimulation index (S.I.) where the numbers represent the mean number of MCp/lung or MCp/10^6^MNC of sensitized FcRγ^−/−^ mice challenged with IgE-anti-TNP/OVA-TNP divided by the mean number obtained in FcRγ^−/−^ mice challenged with OVA-TNP alone. The mean of the S.I. from all experiments is given in bold text at the bottom. The representative experiment shown in (A–B) is indicated in bold italics. There was no statistical difference (ns) in the tested parameters using a two-way ANOVA from a comparison of all individual mice from each group from the three experiments.

The common Fc-receptor γ chain (FcRγ) is required for signaling by FcεRI, FcγRI, FcγRIII and FcγRIV [13], [18]. All of these except FcγRI can bind IgE as an immune complex [14], and we wanted to find out if the enhancement of lung MCp seen after IgE immune complex challenge was dependent on a FcRγ-associated receptor. To this end, wild type and FcRγ^−/−^ mice were OVA sensitized and challenged with IgE-anti-TNP/OVA-TNP. The number of MCp/lung in FcγR^−/−^ mice was less than half of that in wild type mice (e. g. 368±89 versus 893±306, Figure 5A). The lung MCp frequency was also reduced in sensitized IgE-anti-TNP/OVA-TNP challenged FcγR deficient mice compared to wild type mice (Figure 5B). In summary, there was 2.4 times more MCp/lung (p<0.01) and 1.8 times more MCp/10^6^ MNC (p<0.05) in IgE-anti-TNP/OVA-TNP challenged wild type than in FcRγ^−/−^ mice (Figure 5C). These findings implied that IgE-anti-TNP/OVA-TNP could not enhance MCp recruitment in FcRγ^−/−^ mice. However, the low response to IgE immune complexes in FcRγ^−/−^ mice does not *per se* prove that IgE cannot enhance the number of lung MCp in FcRγ^−/−^ mice. A possibility is that FcRγ^−/−^ mice challenged with OVA-TNP alone have an even lower level of lung MCp and that the levels seen in IgE-anti-TNP/OVA-TNP challenged FcRγ^−/−^ mice are nevertheless enhanced. Therefore, sensitized FcRγ^−/−^ mice were challenged with IgE-anti-TNP/OVA-TNP or OVA-TNP alone. Both groups had similar frequency and total numbers of MCp (Figure 6). Thus, IgE immune complexes did not enhance lung MCp in FcγR^−/−^ mice. Altogether, our observations suggest that the enhancement of lung MCp caused by IgE immune complexes is mediated by an FcRγ-associated receptor.

## Discussion

Germinal center formation and production of allergen specific IgE locally in the lung takes place after airway challenge [27] together with the appearance of increased numbers of mature mast cells [1], [2]. Previous studies show that the mechanisms that govern the low basal homing of MCp and the antigen-induced recruitment of MCp to lung differ and suggest that the mast cell hyperplasia is preceded by recruitment of MCp [3], [5], [25]. We hypothesized that formation of IgE immune complexes locally in the lung enhances the MCp recruitment. To test this, IgE immune complexes or antigen alone were administered intranasally to antigen-sensitized mice (Figure 1). Despite the low influx of MCp with intranasal challenge of antigen alone, the same low dose of antigen in complex with IgE stimulated the increase of three times more MCp per mouse lung (Figure 3A, D). This effect was neither due to differences in the levels of endogenously produced antigen-specific IgE (OVA-specific IgE levels in serum were similar between the groups, results not shown) nor to impurities in the antibody preparation, as challenge with anti-TNP IgE alone did not enhance the number of lung MCp (Figure 3A, C). OVA-TNP (70 µg) in itself does not induce higher number of lung MCp over the basal homing seen in naïve or only sensitized mice (Figure 2A–D, [5], [25]). Therefore it is remarkable that specific IgE together with this suboptimal antigen dose is able to induce a 2.1-fold increase in MCp/10^6^ MNC and a 3-fold increase in total MCp content (Figure 3D). Although we do not provide experimental evidence for that the increase in lung MCp is due to recruitment as opposed to increased proliferation of lung MCp *in situ*, we favor the view that the increase in lung MCp is due to recruitment because of the limited time (three days) of treatment with IgE immune complexes. Moreover, mice challenged with IgE immune complexes and IgE alone have encountered the same total amount of IgE. Nevertheless, IgE immune complex challenge stimulated 3-fold more lung MCp whereas IgE alone failed to increase lung MCp (Figure 3A–C). Therefore at least increased proliferation of MCp mediated by IgE is unlikely. Since mature mast cells are excluded from the MNC preparation we can exclude that the increase of lung MCp are due to proliferation of lung mast cells.

Recently, Mathias *et al* found that wild type mice had higher number of mature mast cells in trachea, bronchus and spleen than IgE deficient mice after three weeks of *Aspergillus* treatments [28]. Despite the lack of IgE, the increased level of lung MCp was intact in the IgE deficient mice after three weeks of challenge. The increase in airway mast cells in wild types was interpreted as IgE-mediated enhancement of mast cell survival through FcεRI. Presuming that this protocol induced high levels of antigen-specific IgE locally in the lungs leading to formation of IgE immune complexes in the wild type mice, IgE immune complex mediated enhancement of MCp recruitment could have taken place earlier as IgE levels rise already after seven days. It is also possible that the IgE immune complex enhancement of MCp recruitment only occurs at lower antigen doses.

IgE-mediated immune reactions are both positively and negatively regulated by the low-affinity receptor for IgE, CD23 [16], [29]. Administration of small protein antigens in complex with specific IgE enhances the antibody responses by a 100-fold and T-cell proliferation by 10-fold as compared to administration of antigen alone [17], [21], [30]. These effects are seen only at low doses of antigen and are completely dependent on CD23, and it therefore seemed possible that the enhanced recruitment of MCp could also be mediated by CD23. However, sensitized and IgE immune complex challenged CD23^−/−^ mice had similar lung MCp numbers as wild type mice treated in parallel, thus excluding a major involvement of this receptor (Figure 4).

Other known IgE-binding receptors are FcεRI, FcγRII, FcγRIII, and FcγRIV [13], [18]. Of these, all except FcγRII use the common FcRγ chain for signaling [18]. The number and frequency of lung MCp in sensitized, IgE immune complex challenged FcRγ^−/−^ mice was significantly reduced as compared to that in wild type mice (Figure 5). This finding was further corroborated by the fact that antigen specific IgE did not enhance the lung MCp content in FcRγ^−/−^ mice: similar numbers of MCp were found in FcRγ^−/−^ mice challenged with IgE immune complexes as in FcRγ^−/−^ mice challenged with antigen alone (Figure 6). Thus, our results suggest that the enhanced level of lung MCp after challenge with IgE immune complexes is dependent on an FcRγ-chain-associated receptor, i.e. FcεRI, FcγRIII, or FcγRIV. IgE immune complexes of allotype a, and possibly allotype e, bind FcγRIII [12], [14], [31] whereas IgE immune complexes of allotype b (which is used in our study) do not bind to this receptor [14]. Therefore, it is unlikely that FcγRIII is involved in MCp recruitment, and only FcεRI and FcγRIV remain as candidate receptors.

FcγRIV is a recently discovered Fc-receptor for IgG [13], [14], which also has intermediate affinity for IgE immune complexes (Ka≅1.4–7.5×10^5^ M^−1^ depending on the study and the source of IgE). Since an FcγRIV knockout mouse strain is not available, conclusions about the *in vivo* role of IgE binding to this receptor was based on comparisons between FcRγ^−/−^ mice and quintuple knockout mice lacking FcγRI/II/III, FcεRI, and CD23. The authors concluded that IgE immune complex-induced infiltration of polymorphonuclear cells to the lung was caused by alveolar macrophages expressing FcγRIV in synergy with lung mast cells expressing FcεRI [14]. This effect was only seen after pre-treatment with supernatants from activated mast cells. Since mast cells do not express FcγRIV [13], this may suggest that FcεRI has a superior biological role over FcγRIV to mediate IgE immune complex effects.

FcεRI binds IgE immune complexes with high affinity (Ka≅10^9^–10^10^ M^−1^) [14], [29], [32] and thus has 1000–70 000-fold higher affinity for IgE immune complexes than FcγRIV. For this reason, FcεRI is more likely to be involved in IgE immune complex mediated enhancement of lung MCp numbers than FcγRIV. Indirect support for FcεRI also comes from comparisons between our finding on eosinophils in bronchoalveolar lavage (described below) and a study by Zuberi *et al.* They showed that bronchoalveolar lavage from mice challenged with *in vitro* formed IgE-complexes had higher levels of IL-4 and more eosinophilia than mice challenged with antigen alone and that the effect was dependent on FcεRI [26]. As eosinophils are excluded from the lung MNC preparations, eosinophils were generally not studied in our experiments. However, in one experiment bronchoalveolar lavage was analyzed as described in the results and similar to Zuberi and colleagues, we found that sensitized mice challenged with IgE immune complex had more eosinophilia than mice challenged with antigen alone. This analogy is compatible with the idea that FcεRI is the receptor mediating the enhancement of the number of lung MCp by IgE immune complexes in our system.

Altogether, the ability of IgE immune complexes to enhance the number of MCp in the lung is a new way for IgE to influence mast cells and thereby the allergic response. The results suggest that formation of IgE immune complexes locally in the lung enhances the lung MCp number through an FcRγ associated receptor. The effect is most likely mediated via activation of mast cells and basophils through FcεRI, although a combined effect of IgE immune complexes binding to FcγRIV on other lung cells, such as alveolar macrophages, cannot be excluded. This mechanism is likely important in patients with allergic asthma as these usually have high levels of allergen-specific IgE that may form IgE immune complexes with the specific allergen in their lungs. Our results also suggest that IgE immune complex-mediated enhancement of lung MCp numbers is an additional target for treating patients with anti-IgE (omalizumab).
